# Best Linear Unbiased Prediction of Genomic Breeding Values Using a Trait-Specific Marker-Derived Relationship Matrix

**DOI:** 10.1371/journal.pone.0012648

**Published:** 2010-09-09

**Authors:** Zhe Zhang, Jianfeng Liu, Xiangdong Ding, Piter Bijma, Dirk-Jan de Koning, Qin Zhang

**Affiliations:** 1 Key Laboratory of Animal Genetics and Breeding of the Ministry of Agriculture, College of Animal Science and Technology, China Agricultural University, Beijing, China; 2 The Roslin Institute and Royal (Dick) School of Veterinary Studies, University of Edinburgh, Roslin, United Kingdom; 3 Animal Breeding and Genomics Centre, Wageningen University, Wageningen, The Netherlands; Aarhus University, Denmark

## Abstract

**Background:**

With the availability of high density whole-genome single nucleotide polymorphism chips, genomic selection has become a promising method to estimate genetic merit with potentially high accuracy for animal, plant and aquaculture species of economic importance. With markers covering the entire genome, genetic merit of genotyped individuals can be predicted directly within the framework of mixed model equations, by using a matrix of relationships among individuals that is derived from the markers. Here we extend that approach by deriving a marker-based relationship matrix specifically for the trait of interest.

**Methodology/Principal Findings:**

In the framework of mixed model equations, a new best linear unbiased prediction (BLUP) method including a trait-specific relationship matrix (TA) was presented and termed TABLUP. The TA matrix was constructed on the basis of marker genotypes and their weights in relation to the trait of interest. A simulation study with 1,000 individuals as the training population and five successive generations as candidate population was carried out to validate the proposed method. The proposed TABLUP method outperformed the ridge regression BLUP (RRBLUP) and BLUP with realized relationship matrix (GBLUP). It performed slightly worse than BayesB with an accuracy of 0.79 in the standard scenario.

**Conclusions/Significance:**

The proposed TABLUP method is an improvement of the RRBLUP and GBLUP method. It might be equivalent to the BayesB method but it has additional benefits like the calculation of accuracies for individual breeding values. The results also showed that the TA-matrix performs better in predicting ability than the classical numerator relationship matrix and the realized relationship matrix which are derived solely from pedigree or markers without regard to the trait. This is because the TA-matrix not only accounts for the Mendelian sampling term, but also puts the greater emphasis on those markers that explain more of the genetic variance in the trait.

## Introduction

With the advances in molecular biotechnology, genome-wide high-density single nucleotide polymorphisms (SNP) marker data is becoming available for many farm animal and plant species. These data combined with phenotypic data can be used to estimate genetic merit [1] or predict phenotypic values [2] for the trait of interest. This method was termed genomic selection by Meuwissen *et al.* [1]. In the usual implementation of genomic selection, effects of whole-genome high-density markers are first estimated using a training population in which all individuals are both phenotyped and genotyped. Then, selection candidates that are only genotyped get their genomic estimated breeding values (GEBVs) by adding up all the marker effects estimated from the training population. The greatest advantage of this approach is the predicting ability with potential high accuracy and the possibility to shorten the generation interval by estimating accurate breeding values early in life, even before birth [1], [3], [4]. As a result, genomic selection could save up to 92% of costs for dairy cattle breeding companies [5]. This has led to a rapid development of research and application of genomic selection in animal [5]–[7], plant [8], [9] and aquaculture breeding [10], [11].

In the framework of genomic selection, many statistical methods have been proposed to estimate the marker effects in the training population. Based on the assumptions about the statistical distribution of the marker effects, these methods can be classified into two groups. The first group assumes that all markers have some effect on the trait of interest and that the variance of each marker effect is equal. A typical method using this assumption is ridge regression best linear unbiased prediction (RRBLUP) [1], [12]. The second group allows marker effects to come from different statistical distributions. These methods, sometimes coined ‘variable selection methods’ include BayesA, BayesB [1], Bayesian shrinkage [13] and several others [14]–[18]. The performance of both groups of methods has been compared extensively [1], [3], [19]–[21].

An alternative to estimating GEBVs by summing up all the marker effects, is to estimate GEBVs directly within the framework of mixed model equations (MME). Conventional ‘animal model’ BLUP has been routinely applied in animal, tree and plant breeding for many decades. The predicting ability for individuals without phenotypic records of this method depends on the structure of the random effect variance-covariance matrix. In the classical MME, a numerator relationship matrix (NRM) based on the pedigree [22] is used to describe the additive variance-covariance relationship between all individual pairs in a population. The elements in NRM are twice the expected probabilities that two alleles randomly sampled from the same locus in two individuals are identical by descent (IBD). In recent years, with the availability of more and more genetic markers covering the whole genome, the NRM could be replaced by a realized relationship matrix (RRM) or marker-derived relationship matrix [23], [24].

Current implementations of the RRM are based on the ‘infinitesimal model’ [25], [26], which assumes that a very large number of genes that are evenly distributed across the genome contribute equally to the trait of interest. This assumption is also implicit when using RRBLUP to estimate GEBV. In the framework of genomic selection, the method to estimate GEBVs using the RRM is termed GBLUP, which was shown to be theoretically equivalent to RRBLUP [20], [26]–[29]. Because current NRM and RRM are based on expected or realized average genome-wide information only, they are identical for all traits in a population. However, in animal or plant breeding programs, investigators are interested in the improvement of one or several specific traits. The true genetic architecture for any trait deviates from the infinitesimal model to a certain degree, and different traits are controlled by different sets of genes. Quantitative trait loci (QTL) mapping studies have shown that most quantitative traits are affected significantly by a finite number of genes [30], which are neither evenly distributed nor equally contributing to the trait of interest. When the genetic control of these traits deviates from the assumptions of the infinitesimal model, neither NRM nor RRM including averaged information optimally describes the variance-covariance structure between individuals for the trait of interest. Therefore, it is more realistic to accommodate the departure from the infinitesimal model while constructing the variance-covariance matrix. The genome-wide SNP information provides a tool to assess the genetic architecture of the traits of interest and improve upon NRM and RRM. This possibility has not yet been explored in the framework of MME.

Here we introduce a two-step BLUP method, named ‘best linear unbiased prediction with trait-specific marker derived relationship matrix’ (TABLUP), to estimate GEBVs utilizing trait-specific marker information. A simulation study was performed to investigate the benefit of the presented method for the accuracy of estimated breeding values. The rules to construct the TA matrix were derived. Genomic selection using TABLUP was compared with RRBLUP, BayesB and GBLUP in a range of scenarios. Factors affecting the TABLUP method and its features were discussed.

## Materials and Methods

Our method involves two steps. First, the SNP effects in the training population, in which all individuals have their genotypic and phenotypic data available, are estimated using one of the methods mentioned above. Then, a trait-specific relationship matrix (TA) was derived from all the marker genotypes and their weights obtained from the first step. Finally, GEBVs of genotyped individuals, including all phenotyped individuals and other young non-phenotyped individuals, was estimated using MME with the TA-matrix.

### Estimation of the marker effects

Any method that has been proposed in the framework of genomic selection can be used to estimate marker effects in the training population. In our study, RRBLUP and BayesB were used with the following statistical model:
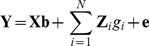
(1)where **b** is a vector of fixed effects (including an overall mean), *g_i_* is the *i^th^* marker effect, *N* is the total number of markers, **X** and **Z** are design matrices corresponding to **b** and *g*, and **e** is a vector of residual errors. In design matrix **Z**, for SNP markers with alleles 1 and 2, genotypes were represented as 0, 1 and −1 to denote the heterozygous (12) and the two homozygous genotypes (22 and 11), respectively. We assume that the residuals are independent and follow a normal distribution, **e**∼N(0, **I**σ*_e_* ^2^). All marker effects *g_i_* are also assumed to be normally distributed, *g_i_*∼N(0, σ*_gi_* ^2^), where σ*_gi_* ^2^ is the variance of effect of marker *i*, The σ*_gi_* ^2^ is assumed to be equal for all markers in RRBLUP and variable in BayesB for which a marker may have a variance of zero with a probability of *π* or a variance following a scaled inverse chi-square distribution with 1 degree of freedom with a probability of 1−*π* [31].

In the RRBLUP method, the simulated variance components were used as the true variance in the analyses. The *i^th^* marker variance was calculated from σ*_gi_* ^2^ = σ*_a_* ^2^/*N*, whereσ*_a_* ^2^ is the total additive genetic variance, as proposed by Meuwissen *et al.* [1].

In the BayesB method, the exact ratio of the number of simulated QTL to the total number of markers was used as the prior value of 1−*π*. The Monte Carlo Markov chain (MCMC) algorithm of BayesB is a mixture of Gibbs sampling and Metropolis-Hastings sampling as described by Meuwissen *et al.* [1]. In our research, the MCMC chain was run for 10,000 cycles with 100 cycles of Metropolis-Hastings sampling in each Gibbs sampling, and the first 2,000 cycles were discarded as burn-in. All the samples of marker effects from later cycles were averaged to obtain the estimates of marker effects.

### Construction of trait specific marker derived relationship matrix (TA)

A relationship matrix constructed using all markers without trait-specific weighting is equivalent to the G matrix in GBLUP, the so-called realized relationship matrix, and is identical for all traits. The trait-specific relationship matrix, in contrast, should specify the genetic covariance between two individuals for the trait of interest. The contribution of each locus to this covariance consists of two components: the IBD between both individuals, which is reflected by their marker genotypes, and the contribution of the locus to the genetic variance in the trait. Thus elements of the TA-matrix were obtained as
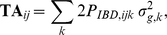
where 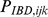 is the IBD-probability at locus *k* between individual *i* and *j*, 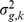 is the contribution of locus *k* to the genetic variance in the trait, and the sum is taken over all loci. This expression ignores covariances of genetic effects at different loci, which may originate e.g. from linkage disequilibrium.

To simplify the arithmetic, we first obtained the full identity by state (IBS) at each locus. Subsequently we calculated the weighted average IBS over all loci, and finally corrected for the population average IBS to obtain a mean relatedness equal to zero. The full IBS at locus *k* between individual *i* and *j* is calculated as [23], [32]

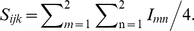
(2)where *I_mn_* is 1 if allele *m* in the first individual is identical to allele *n* in the second individual or 0 otherwise. All four possible combinations are taken into consideration in formula (2). Therefore, the genotype data does not need to be phased. Next, the weighted average IBS for individuals *i* and *e*, taking all markers into account, was obtained as
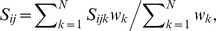
where *N* is the total number of loci and *w_k_* is the weight for the *k^th^* marker. In the present study, we compared two different weights, weights were either equal to the posterior variances of marker effects estimated from BayesB (denoted TAB), or weights were equal to the expected variances of marker effects derived from RRBLUP (denoted TAP). For RRBLUP, the expected variance for the *k^th^* marker was calculated as 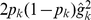, where the *p_k_* is the frequency of one allele on that locus and 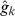 is the estimated marker effect. Then we corrected for the mean IBS, using an adjustment based on Wright's F-statistics
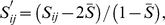
where 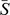 is the population average of *S_ij_*. Finally, science relatedness equals twice the IBD, we obtained elements of the marker-based relationship matrix as
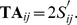
An overview of different methods to construct the TA matrix is shown in Table 1.

**Table 1:** Overview of different methods to construct the trait-specific relationship matrix.

Acronym	Estimation^a^	Weighted
TAP	RRBLUP	Yes
TAB	BayesB	Yes
GBLUP	–	No

a The method used to estimate the marker effects.

### Estimation of genomic breeding values

For TABLUP, the GEBVs of all genotyped individuals are predicted by solving the MME, which included the TA matrix. The statistical model was
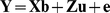
(3)where ***u*** is the random polygenic effect, which is the EBV in conventional BLUP and GEBV in GBLUP or TABLUP. The solution for ***u*** is equal to 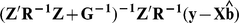, where **G** is the **TA** matrix for the TABLUP method that was inverted numerically. The simulated variance components were provided to the MME, which were solved by Gauss-Seidel iteration.

For RRBLUP and BayesB, the GEBV of a genotyped individual was calculated as the sum of all estimated marker effects according to its marker genotypes [1].

### Data simulation

**Figure 1:**
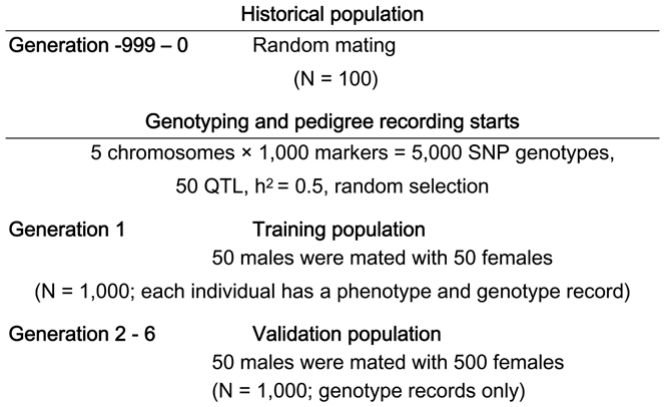
Overview of the simulated population structure.

The simulation started with a base population of 100 individuals, followed by 1,000 non-overlapping generations with the same population size, denoted as generation −999 to generation 0 to indicate historical generations. In the base population and each historical generation, 50 males were randomly mated with 50 females and each mating produced two offspring (one male and one female). After the 1,000 historical generations, six additional generations, numbered 1 to 6, were simulated. In generation 1, the population size was expanded from 100 to 1,000 by randomly mating 50 males with 50 females from generation 0, where each female produced 20 progeny (10 males and 10 females). From generation 1 to 5, 50 males were randomly selected from the 500 male individuals to be the sires of the next generation, and all 500 females were used as dams without selection. The population size of 1,000 for generation 2 to 6 was obtained by randomly mating each male with 10 females and each female produced two offspring. This resulted in a half sib family structure as depicted in Figure 1.

The simulated genome consisted of five chromosomes with a total length of 5 Morgan (1 Morgan per chromosome). On each chromosome, 1,000 marker loci were randomly located and each segment between two markers was considered to harbor a potential QTL, giving 5,000 markers and 4,995 potential QTL in total. Based on the distance between two adjacent loci, Haldane's mapping function was used to calculate the probability of having a recombination between adjacent loci on the same chromosome.

**Figure 2:**
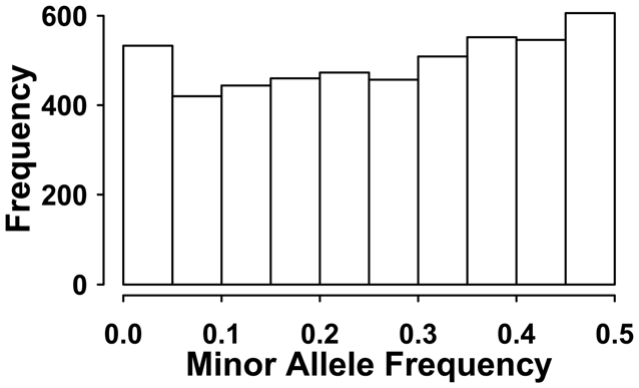
The typical distribution of minor allele frequency of the simulated genotypic data.

The mutation-drift equilibrium model was used to create polymorphic markers and QTL. In the base population, all markers and QTL had both alleles coded as 1. Mutations were allowed in all historical generations for all loci with a mutation rate of 1.25×10^−3^ per locus, per generation, and per animal. Under the mutation-drift equilibrium model, the expected heterozygosity when the population reaches equilibrium is 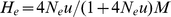, where *Ne* is the effective population size and *u* is the mutation rate [33]. Therefore, the proposed mutation rate gave an expected heterozygosity of 0.5. For each new mutation on the same locus, a unique allele was created and coded with a new number sequentially starting from 2. In generation 0, recoding of alleles was implemented to obtain bi-allelic SNP markers. For each locus, the allele that had a frequency closest to 0.5 was recoded as 1, while all other alleles were recoded as 2 following Solberg *et al.* [34] while differing from the rule used by Meuwissen *et al.* [1], in which only part of the putative loci were polymorphic and available for data analysis. The distribution of minor allele frequencies of our simulated data can be seen in Figure 2.

For each individual from generation 1 to 6, a true breeding value (TBV) was simulated by summing up all true QTL genotypic values, i.e., 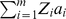, where *a_i_* is the allele substitution effect of the *i^th^* QTL, and *Z_i_* is 0, 1, or −1 corresponding to genotypes 12, 22 and 11, respectively. In our standard scenario, 50 QTL were randomly selected from the 4,995 putative QTL. For each true QTL, the allele substitution effect *a*_i_ was drawn from a gamma distribution with the shape parameter 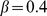 and scale parameter 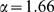. The allele substitution effect *a_i_* sampled from a gamma distribution may be positive or negative with equal probability, following Meuwissen *et al.* [1].

The total genetic variance was computed as the sum of variances across all QTL with the assumption of no correlation between QTL. The simulated additive genetic variance of each QTL was calculated as 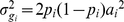 [35], where *p_i_* is the allele frequency at the *i* ^th^ QTL in generation 1, and *a_i_* is the allele substitution effect of the *i* ^th^ QTL. The allele substitution effects were re-scaled to have a total additive genetic variance (σ*_A_* ^2^) of 1.

Only the 1,000 individuals in generation 1 were assigned a phenotypic record. The phenotypic value *P_i_* of the *i^th^* individual was obtained by 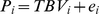, where *e_i_* is randomly sampled from a normal distribution N(0, σ*_e_* ^2^). The environmental variance, σ*_e_* ^2^, equaled 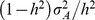. In our standard scenario, heritability was set to be 0.5, so that environmental variance was 1. Breeding values of individuals without phenotypic records were predicted using Equation 3. The accuracy of predicted breeding values was evaluated by calculating the correlation between the true and predicted breeding values for individuals without phenotypic records.

To investigate the effect of number of QTL and heritability on the accuracy of GEBVs, two groups of alternative scenarios were simulated in addition to the standard scenario described above. In the first group, four different levels of heritability were simulated: 0.05, 0.1, 0.3 and 0.9. In the second group, different numbers of QTL were simulated: 100, 200, 500 and 1,000. For all these alternatives, only the intended parameter was altered from the standard scenario. For all scenarios, 10 replicates were simulated.

## Results

### Estimates of QTL effects

**Figure 3:**
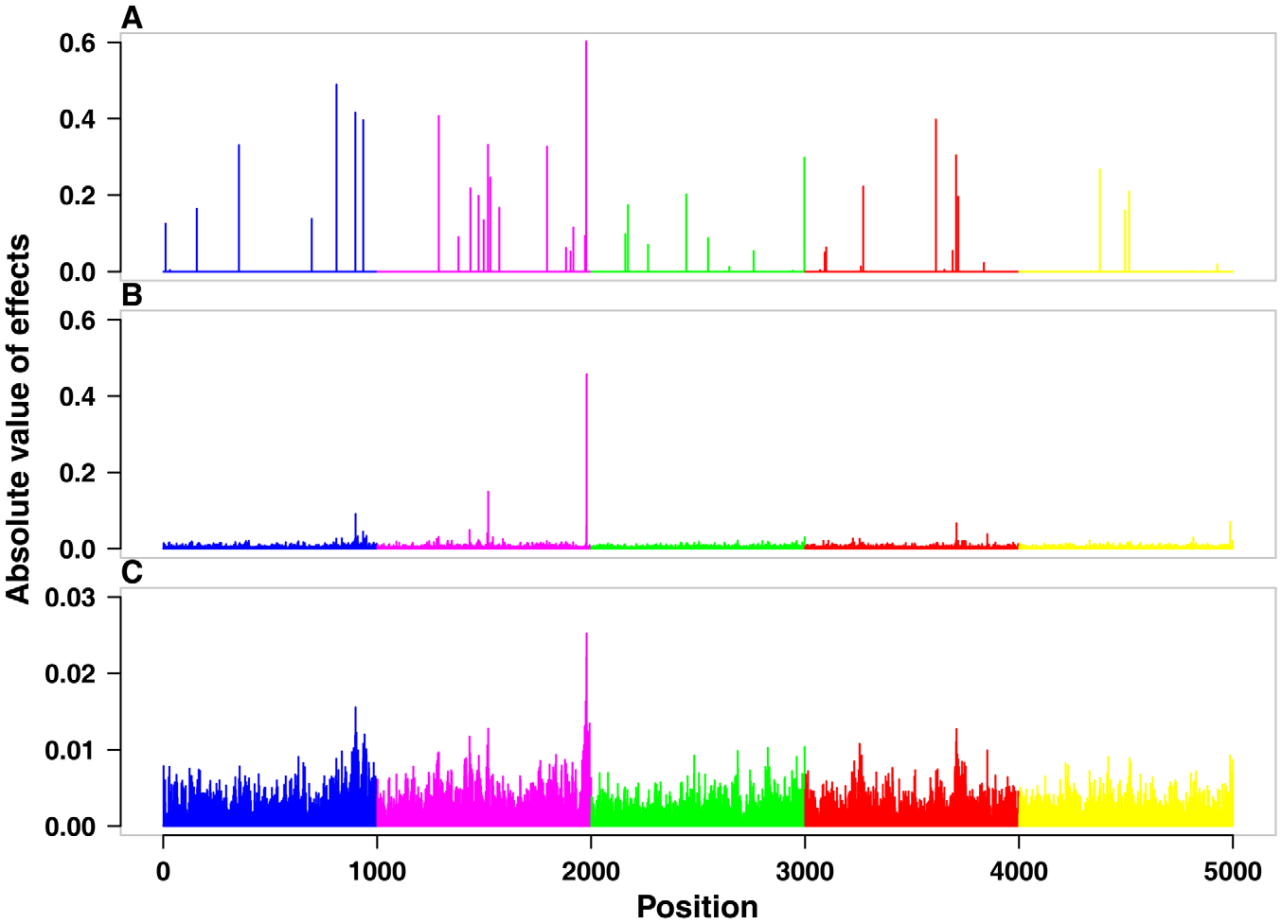
True and estimated QTL effects from a randomly selected replicate. Panel A shows the absolute values of the simulated true QTL effects throughout the simulated genome. Panel B shows the absolute estimates of the marker effects throughout the genome use the BayesB approach. Panel C shows the absolute estimates of the marker effects throughout the genome use the RRBLUP approach. There were 50 true QTL and 5,000 markers. Beware of the scale difference in panel C.

The simulated (true) QTL effects and the marker effects estimated from RRBLUP and BayesB from one random replicate of the standard scenario are shown in Figure 3. While the simulated absolute QTL effects ranged from 0 to 0.6 (Figure 3A), the estimated absolute marker effects ranged from 0 to 0.5 for BayesB (Figure 3B) and 0 to 0.025 for RRBLUP (Figure 3C; beware of the difference in scale between Figures 3A, B and C). Most segments containing big QTL were mapped by both methods. However the resolution of BayesB was higher than that of RRBLUP.

### Pearson correlation, rank correlation and regression coefficient

Table 2 shows the Pearson correlations and rank correlations between the predicted breeding values (GEBVs) and the simulated true breeding values (TBVs) as well as the regression of TBVs on GEBVs in generation 2. In terms of accuracy, which is defined as the Pearson correlation between GEBVs and TBVs, both TABLUP methods (TAB and TAP) performed better than RRBLUP and GBLUP. TAB performed better than TAP but a little worse than BayesB. However, the difference between TAP and TAB (0.042) was much smaller than that between RRBLUP and BayesB (0.085). In other words, the TABLUP appears to be less sensitive to the genetic architecture than either RRBLUP or BayesB.

**Table 2:** Correlation and rank correlation between estimated and true breeding values as well as regression of true breeding values on estimated breeding values in generation 2 (*N_qtl_* = 50, *h^2^* = 0.5).

Method	Correlation	Rank correlation	Regression
BayesB	0.809±0.009	0.798±0.010	0.998±0.014
RRBLUP	0.724±0.011	0.710±0.011	1.064±0.015
TAP	0.748±0.010	0.736±0.010	0.949±0.015
TAB	0.790±0.008	0.778±0.009	0.899±0.016
GBLUP	0.726±0.012	0.712±0.011	0.997±0.015

In breeding practice, rank correlation is more important than Pearson correlation, especially in truncation selection. On average, the rank correlation is 0.013 lower than the Pearson correlation. The ranking of the methods and the trend of both correlations were the same (Table 2).

The regression coefficient of TBVs on GEBVs was used to measure the biases of GEBVs from different methods (Table 2). RRBLUP and BayesB gave almost unbiased estimates of GEBVs, while both TABLUP methods slightly underestimated GEBVs.

It is notable that GBLUP and RRBLUP performed equally in terms of correlations, which confirms the theoretical equivalence of the two methods. However, the regression coefficient was slightly different between these two methods (Table 2).

### Decline of accuracy over generations

**Figure 4:**
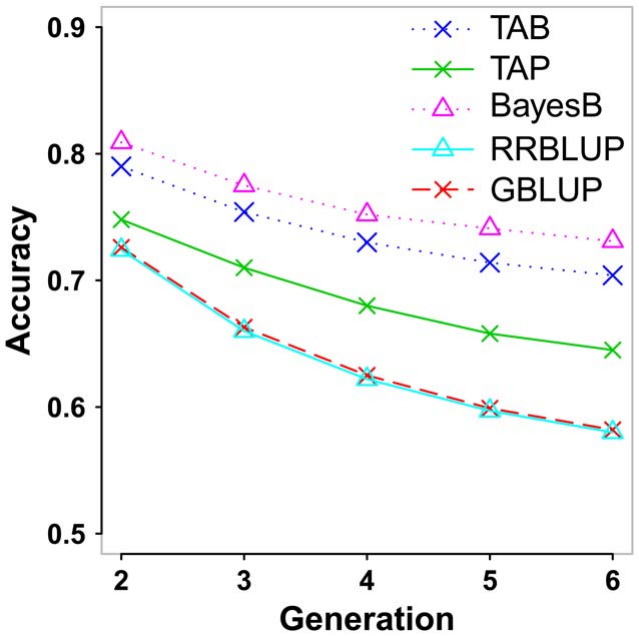
Accuracy of genomic breeding values (GEBVs) using 5 different approaches. The graph shows the correlation between estimated and true breeding values in generations 2–6 using GEBVs derived by a variable selection approach (BayesB), an approach using infinitesimal model (RRBLUP), BLUP methods with the trait-specific matrix using BayesB weights (TAB), the trait-specific matrix using infinitesimal model weights (TAP) and the average genomic relationship matrix using the infinitesimal model (GBLUP).

The decline of accuracy of GEBVs over generations can be a measure of the persistency of the predicting ability for different methods. As shown in Figure 4, the average decreases in accuracy per generation from generation 2 to 6 were 0.021 and 0.026 for TAB and TAP, and 0.020 and 0.036 for BayesB and RRBLUP, respectively. Due to the high persistency of TAP, the advantage of TAP over RRBLUP in accuracy increased from 0.016 in generation 2 to 0.065 in generation 6. Again, GBLUP showed the same decline pattern as RRBLUP.

### Effect of number of simulated QTL

With the increase of the number of simulated QTL from 50 to 1,000, the accuracy of GEBVs in generation 2 decreased consistently for BayesB, increased consistently for RRBLUP and GBLUP (except for the case of 200 QTL), and decreased first (from 50 to 200 QTL) and then increased (from 200 to 1000 QTL) for both TABLUP methods, as shown in Table 3. A general tendency is that the differences between different methods reduced along with the increase of the number of QTL. It seems that BayesB is more sensitive to the number of QTL than the other methods, in particular when the number of QTL increased from 50 to 200. Therefore, the advantage of BayesB over the other methods decreased with the increase of number of QTL.

**Table 3:** Accuracy of GEBVs for different simulated QTL numbers in generation 2 (*h^2^* = 0.5).

Number of QTL	BayesB	RRBLUP	GBLUP	TAB	TAP
50	0.809±0.009	0.724±0.011	0.726±0.012	0.790±0.008	0.748±0.010
100	0.786±0.012	0.740±0.017	0.739±0.017	0.770±0.013	0.744±0.015
200	0.763±0.011	0.734±0.012	0.735±0.011	0.749±0.010	0.724±0.010
500	0.763±0.009	0.745±0.009	0.748±0.010	0.756±0.010	0.732±0.009
1000	0.760±0.010	0.756±0.012	0.756±0.012	0.755±0.012	0.736±0.012

### Effect of heritability

Table 4 shows the accuracies of GEBVs for different methods while varying the heritability. By decreasing the heritability from 0.9 to 0.05, the accuracies of all methods decreased as expected. Again, TAB performed slightly worse than BayesB but better than all other BLUP-type methods in all cases, although its advantage declined with the decrease of heritability.

**Table 4:** Accuracy of GEBVs for different heritability in generation 2 (*N_qtl_* = 50).

Heritability	BayesB	RRBLUP	GBLUP	TAB	TAP
0.05	0.407±0.020	0.376±0.021	0.374±0.020	0.394±0.018	0.354±0.019
0.1	0.542±0.023	0.472±0.017	0.472±0.018	0.518±0.015	0.464±0.017
0.3	0.735±0.015	0.638±0.014	0.641±0.014	0.708±0.011	0.656±0.013
0.5	0.809±0.009	0.724±0.011	0.726±0.012	0.790±0.008	0.748±0.010
0.9	0.908±0.004	0.861±0.006	0.862±0.006	0.910±0.004	0.886±0.005

## Discussion

The main aim of this study was to present the two-step TABLUP method, which utilizes a trait-specific relationship matrix (TA) in the mixed model equations (MME), for estimating genomic breeding values in the framework of genomic selection. Rules to construct the TA matrix were derived and implemented. The performance of the TABLUP method was shown via simulation to compare with several other popular approaches under different scenarios.

The trait-specific relationship matrix TA is related to the trait of interest by including the information of both marker genotypes and the marker effect variances. In terms of predicting ability, the proposed TA matrix is an improvement upon the classical numerator relationship matrix (NRM) and the realized relationship matrix (RRM). In the framework of MME, the conventional BLUP, GBLUP and TABLUP use NRM, RRM and TA matrix as variance-covariance matrix for random genetic effects, respectively. The advantage of RRM over NRM has been investigated previously [24]–[27]. This advantage results from the fact that RRM captures the Mendelian sampling deviations, which accounts for half the additive genetic variance among individuals [20], [25], [28], [36]. We infer that the advantage of using the TA matrix over RRM and NRM is because it not only accounts for the Mendelian sampling term, but also puts greater weight on loci explaining more of the genetic variance in the trait.

The comparable performance of TABLUP and BayesB, especially between TAB and BayesB, suggests that TABLUP might be an equivalent model of BayesB. The equivalence between GBLUP and RRBLUP has been proven under the assumption that all markers contribute equally to the trait of interest [20], [26]–[29]. Whether the same equivalence exists between TABLUP and BayesB is an interesting hypothesis, but outside the scope of this manuscript. However, as the TA matrix can take the trait-specific genetic architecture into consideration, the performance of TABLUP should be more robust with respect to the genetic architecture of the trait of interest. The effect of genetic architecture on genomic selection methods has been investigated in detail by Daetwyler *et. al* [31].

TABLUP and GBLUP have some features that other genomic selection methods based on model (1) lack. The most important feature is that the reliability of an individual's GEBV can be calculated. The reliabilities of GEBVs for single individuals are important for breeders to make selection decisions. The calculation of reliabilities using TABLUP is identical to that outlined for GBLUP by VanRaden [28] and Strandén *et al.* [37]. In real data analysis for dairy cattle, this reliability agreed well with the realized reliability [38]. The second feature is that the model for TABLUP could be extended to include non-genotyped individuals. In practice, not all individuals with phenotypic record(s) or reliable EBV(s) can be genotyped. To estimate GEBVs using BayesB or RRBLUP, it is required that all individuals are genotyped. However, this might not be the case for TABLUP and GBLUP. This extension was introduced by Legarra *et al.* [39], who proposed a rule to construct a joint pedigree-genomic relationship matrix. A simulation study demonstrated that this extension can increase the accuracy due to a larger size of training population [40]. Such an extension can also be applied to TABLUP based on model (3) by replacing the TA matrix with a pedigree-TA matrix, so that the non-genotyped individuals can be included in the model and their GEBVs can be estimated. These favorable features should make TABLUP more competitive.

Choosing the right genomic selection method to apply in practical breeding is a challenge for breeders. In simulation studies, BayesB is nearly always better than RRBLUP [1]. In practice, its performance was reported to be nearly equal to or even worse than RRBLUP or GBLUP for some traits [19], [38], [41], [42]. This suggested that the underlying genetic architectures of some traits are closer to the infinitesimal model than expected. However, the data analysis on fat percentage in dairy cattle shows there are single genes like *DGAT1* that may favor the BayesB type approaches [38], [41]. The genetic architectures vary between different traits and for some traits the deviation from the infinitesimal model may be greater than for others. The present study shows that BayesB is more sensitive to the number of QTL underlining a trait than TABLUP and RRBLUP, while the performances of TAB and BayesB are very comparable. Therefore, TABLUP might hold an advantage when applied to real data where the genetic architectures underlining the traits of interest are unknown. However, the performance of TABLUP in practical applications is yet to be evaluated.

In our study, the IBS scoring rule proposed by Eding and Meuwissen [23] was used as a measure of relatedness between individual pairs. It was reported that a singularity problem could arise with some other rules if only a limited number of markers were included into the genomic relationship matrix [28]. For the scenarios presented here, the TA matrix could always be inverted directly without the singularity problems. Moreover, different scoring rules might cause the difference in predicting ability of GBLUP/TABLUP. By setting the diagonal elements of TA matrix as 1 with the assumption of no inbreeding and not removing the IBS for the *S_ij_*, the TA matrix showed a higher predicting ability than that of the current IBS scoring rule (Table 5). Therefore, the effect of different IBS scoring rules to GBLUP/TABLUP still needs to be investigated.

**Table 5:** Accuracy of GEBVs for different rules to construct the relationship matrix in generation 2 (*N_qtl_* = 50).

Heritability	GBLUP	TAB	TAP	TAB^a^	TAP^b^
0.05	0.374±0.020	0.394±0.018	0.354±0.019	0.404±0.021	0.388±0.019
0.1	0.472±0.018	0.518±0.015	0.464±0.017	0.540±0.023	0.497±0.016
0.3	0.641±0.014	0.708±0.011	0.656±0.013	0.734±0.014	0.677±0.014
0.5	0.726±0.012	0.790±0.008	0.748±0.010	0.808±0.009	0.764±0.010
0.9	0.862±0.006	0.910±0.004	0.886±0.005	0.910±0.004	0.884±0.005

a TABLUP with the TA-matrix weighted by the absolute values of estimated marker effects from BayesB and without the correction of the mean IBS.

b TABLUP with the TA-matrix weighted by the absolute values of estimated marker effects from RRBLUP and without the correction of the mean IBS.

The weighting rule used to construct the TA matrix was based on the expected covariance between individuals on the basis of the estimated marker effects. Because this follows the theoretical basis of the relationship matrix this type of weighting should in theory be optimal. However, we cannot exclude that for certain scenarios, other ad-hoc approaches may give a higher accuracy. For example, in Table 5, we show the performance of the weights presented in this manuscript in comparison to using *ad-hoc* weights, which are the absolute estimated SNP effect for BayesB and RRBLUP. We could not find an explanation why these *ad-hoc* weights performed slightly better for the scenarios presented in this study. In this paper, the weights were derived from the marker effect estimation step which increases the computational burden. However, the marker effect estimation step might be not necessary as marker weights could be provided by existing genome-wide association studies (GWAS) or known candidate-gene effects. In such a scenario, SNPs in LD with known mutations could be given weights according to their known effects or variances, while equal weights could be assigned for the remainder of the genome. Likewise, SNPs in known QTL regions could be assigned more informative weights. Also, by setting the weights for non-informative markers to 0, a subset of informative markers could be tested in TABLUP for the purpose of selecting low density markers to reduce the cost of genotyping in selection candidates.

In conclusion, this article introduced the TABLUP approach as a flexible alternative between BayesB and GBLUP. For the scenarios studied, the proposed TABLUP method showed an advantage over GBLUP and RRBLUP, and performed nearly equally to BayesB in terms of accuracy of GEBVs. The TA matrix models both the Mendelian sampling term as well as the genetic architecture underlying the trait of interest. Therefore the application of TABLUP in genomic selection merits further exploration.
